# On the role of tail in stability and energetic cost of bird flapping flight

**DOI:** 10.1038/s41598-022-27179-7

**Published:** 2022-12-31

**Authors:** Gianmarco Ducci, Gennaro Vitucci, Philippe Chatelain, Renaud Ronsse

**Affiliations:** 1 Institute of Mechanics, Materials, and Civil Engineering, UCLouvain, 1348 Louvain-la-Neuve, Belgium; 2 (DICATECh) Dipartimento di Ingegneria Civile, Ambientale, del Territorio, Edile e di Chimica, Politecnico di Bari, Via Re David 200, 70126 Bari, Italy

**Keywords:** Biological physics, Applied mathematics, Aerospace engineering, Animal migration

## Abstract

Migratory birds travel over impressively long distances. Consequently, they have to adopt flight regimes being both efficient—in order to spare their metabolic resources—and robust to perturbations. This paper investigates the relationship between both aspects, i.e., energetic performance and stability, in flapping flight of migratory birds. Relying on a poly-articulated wing morphing model and a tail-like surface, several families of steady flight regime have been identified and analysed. These families differ by their wing kinematics and tail opening. A systematic parametric search analysis has been carried out, in order to evaluate power consumption and cost of transport. A framework tailored for assessing limit cycles, namely Floquet theory, is used to numerically study flight stability. Our results show that under certain conditions, an inherent passive stability of steady and level flight can be achieved. In particular, we find that progressively opening the tail leads to passively stable flight regimes. Within these passively stable regimes, the tail can produce either upward or downward lift. However, these configurations entail an increase of cost of transport at high velocities penalizing fast forward flight regimes. Our model-based predictions suggest that long range flights require a furled tail configuration, as confirmed by field observations, and consequently need to rely on alternative mechanisms to stabilize the flight.

## Introduction

Biological fliers are a source of inspiration for the scientific community. Their capacity to travel over long distances during migrations, their responsiveness to environmental perturbations, and their maneuvering skills are intriguing and inspiring biologists and engineering advances. A particularly outstanding capacity is how they can robustly react to gusts and other perturbations^1,2^. A foundational study by Smith^3^ developed a theory of *evolution of instability*, establishing how inherently unstable flight regimes might have provided a selective advantage for fliers through evolution. Indeed, passively unstable systems are more responsive to changes in command, and this might have facilitated maneuverability for birds. This had to come in parallel with the development of sensory-driven neural circuitries to actively control the flight in order to display stable closed-loop behaviour.

Over the last couple of decades, several studies have investigated how such stability might be achieved, with a specific focus on the gliding regime. Thomas and Taylor^4^ studied gliding flight and showed that birds use a combination of passive stability—alleviating perturbations without active control—governed by their morphology, and active stabilization from neural pathways to regulate their flight. For example, in gliding gulls, static longitudinal stability is achievable by actively modulating the opening of the elbow joint over a large range^5–7^. Cheney et al.^1^ investigated the role of wing compliance and tail actuation in order to alleviate perturbations. Ajanic et al.^8^ conducted a dedicated study on wing morphing and the mechanism of wing sweep on a propelled gliding robot. For each morphological configuration, the authors estimated the required power to fly. They showed that increasing the tail surface was beneficial for longitudinal passive stability, although at the cost of increasing parasitic drag and thus decreasing energetic performance.

Gliding is a flight regime that does not produce the thrust needed to maintain flight altitude over long distances. This is instead possible with the flapping regime. However, studying passive stability of flapping flight requires a dedicated framework to handle the periodic nature of this locomotion regime, and thus the existence of limit cycles instead of fixed equilibrium points. In particular, such a framework must capture the periodic kinematic-dependent nature of aerodynamic forces. Taylor and Thomas^9^ pioneered the development of such a mathematical framework for studying longitudinal stability in flapping flight. They extended the concept of the so-called static margin to flapping flight. They stated that—similarly to gliding—the location of the mean aerodynamic forces with respect to the body center of mass would affect significantly the longitudinal stability. Taylor and Żbikowski^10^ re-defined stability of flapping flight as the asymptotic orbital stability of a limit cycle in the phase space. Following this approach, Dietl and Garcia^11^ used for the first time Floquet theory^12^ to study the phase space limit cycle described by the equations of motion of an artificial flying device, namely an ornithopter. We recently leveraged the same formalism to characterize the passive stability of large migratory birds^13^. Our framework builds upon a morphing quasi-steady lifting line model to compute the aerodynamic forces, and a multiple-shooting algorithm to identify limit cycles. The aerodynamic effects induced by the tail were not captured within this previous work, and the flight was consequently characterized by an unstable mode in pitch whatever the wing configuration.

Moreover, in flapping flight, the wing kinematics has an important impact on power consumption. In an analysis on pigeons, Parslew^14^ suggested that particular kinematics modes might be selected in specific flight regimes for energy-saving purposes. Colognesi et al.^15^ also showed a dependency between power requirement and key parameters of the wing kinematics, specifically the wingbeat amplitude.

Although the role of the tail has been studied in gliding regimes, and the influence of wing kinematics has been studied to assess the performance in flapping regimes, no study to date combined both in a whole body characterization of flapping gaits. The objective of this paper is to provide such a complete modeling of flapping flight that can simultaneously assess the influence of wing kinematics and tail morphology in stability and energetic performance. Such a framework would be of direct relevance to challenge biological hypotheses suggesting an evolution towards passively unstable flight regimes for enhancing manoeuvrability.

Flying animals in the wild can be observed furling their tails, if they possess any, while travelling long distances (e.g., see^16^). This constitutes quite a conundrum for biologists. On the one hand, basic aerospace engineering teaches us that an auxiliary surface aft of the center of mass contributes to static stability. On the other, behavioural biology hints at a cognitive costs entailed by an intrinsically unstable flight regime^3^. To add to the debate, experiments performed on birds flying in wind tunnels over a wide range of speeds have shown that the tail spread significantly decreases with speed, i.e. with increasingly demanding power requirements, both in gliding and flapping regimes^17–19^. This is likely due to a larger wetted area, causing an additional contribution of parasitic drag which, at high velocity, may influence the energetic performance of the flier. These elements are conducive to the hypothesis that tail spreading inherently leads to passively stable flight regimes, at the price of an increased energetic cost. The present work aims at testing that assumption by means of theoretical and numerical frameworks.

Finally, for completing our framework, we need to introduce tail aerodynamics and morphing, whose modelling is a debated topic in literature. In the seminal comprehensive study on tail aerodynamic functions^20^, slender delta wing theory, i.e. the theory for low aspect ratio thin bodies, was prospected as a simplistic though effective model of tail as a lifting surface. This aerodynamic coupling between wings and tail allowed predicting an energetically convenient sequence of quantitative bird morphology modifications as a function of flight speed, specifically wings and tail openings and tail angle of attack under the assumption of minimum power consumption as main gait modulation goal. These considerations were challenged by^19^ via comparisons with in vivo wind tunnel measurements. Whereas in the experiments the total lifting surface, i.e. wings plus tail areas total planform, decreased with airspeed, the quantitative measurements^19^ did not support the estimates of^20^. Birds appear to change morphology more gradually than according to the algorithm set in^20^. One year later, a direct empirical test on the delta wing modelling appropriateness was carried out in^21^. Wind tunnel aerodynamic force production was measured on frozen birds in gliding posture. Tail pitch and spread space was explored and compared to an artificial delta wing. It turned out that the tested tails actually did behave like the artificial delta wing, but the slender wing theory traditionally used for simulating its delta wing like response has limitations. Namely, as for most linearised theories, its fidelity decreases with tail pitch because of stall due to flow separation and must therefore be used with caution. Further evidence about the applicability of the theory was provided by^22^, who measured shed vorticity in the wake of birds in bound posture, i.e. furled wings. Flow visualization techniques demonstrated that the vorticity is concentrated at the lateral tail tips, as predicted by slender wing theory as opposed, for instance, to the lifting line model, in which case a continuous vortex sheet is expected. In that paper it was also confirmed that lift increases monotonically with tail surface. All this considered, it seems widely accepted that spread tails do produce lift and in this work we rely on delta wing theory, but limit tail tilt and spread in order to include the aforementioned considerations.

## Materials and methods

In this section, we introduce flapping flight dynamics and describe the bird model used in our computational framework. Furthermore, we describe how such a dynamical model is used in order to identify steady and level flapping flight regimes, study their stability, and assess their energetic performance.

### Equations of motion modelling flapping flight

Flight dynamics is restricted to the longitudinal plane and thus the bird main body is captured as a rigid-body with three degrees of freedom, i.e. two in translation and one in rotation. This model preserves symmetry with respect to this plane, without any lateral force and moment. The aerodynamic model of the wing relies on the theory of quasi-steady lifting line^23^. Additionally, the present work does not account for the inertial forces due to the acceleration of the wing, and thus also neglecting the so-called inertial power. This inertial power was shown to be negligible in fast forward flight conditions, in comparison to the other contributions to actuation power^24^, and is thus systematically neglected in similar work^10,11,25^ since wing inertia is neglected.

**Figure 1:**
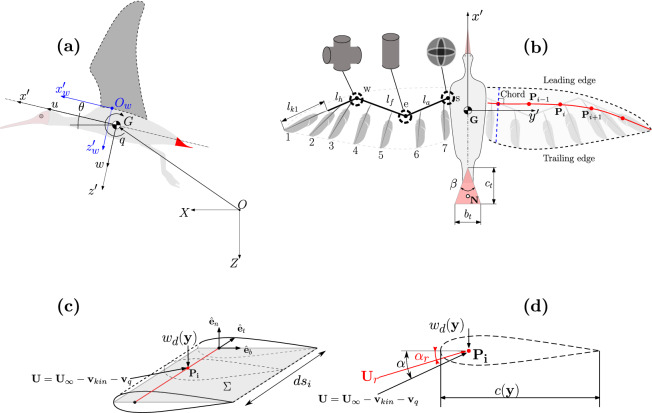
(**a**) Bird model for describing the flight dynamics in the longitudinal plane. The state variables are expressed with respect to the moving body-frame located at the flier’s center of mass $G(x',z')$. These state variables are the component of forward flight velocity, *u*, the velocity component of local vertical velocity, *w*, the orientation of this body-centered moving frame with respect to the fixed frame, $\theta$ and its angular velocity, *q*. A second frame $O(x'_{w}, z'_{w})$ is used to compute the position of the wing, relative to the body. The wings (dark gray) and the tail (red) are the surfaces of application of aerodynamic forces. (**b**) Top view of the bird model. The left wing emphasizes a cartoon model of the skeleton. The shoulder joint **s** connects the wing to the body via three rotational degrees of freedom (RDoF), the elbow joint **e** connects the arm with the forearm via one RDoF and the wrist joint **w** connects the forearm to the hand via two RDoF. Each feather is attached to a bone via two additional RDoF, except the most distal one ”1” which is rigidly aligned with the hand. The right wing further emphasizes the lifting line (red) which is computed as a function of the wing morphing. The aerodynamic forces generated on the wing are computed on the discretized elements $P_{i}$. The tail is modelled as a triangular shape with fixed chord $c_{t}$ and maximum width $b_{t}$ that can be morphed as a function of its opening angle $\beta$. (**c**) Wing element *i* between two wing profiles, identifying a plane $\Sigma$ containing the lifting line (red). (**d**) Cross section of the wing element, containing the chord point $\mathbf {P_i}$ where the velocities are computed (Color figure online).

The body is thus modelled with a mass $m_b$ and a rotational inertia $I_{yy}$ about its center of mass. The equations of motion are expressed in the body frame $G(x', y', z')$ with unit vectors $(\hat{\textbf{e}}_{x'}, \hat{\textbf{e}}_{y'}, \hat{\textbf{e}}_{z'})$, and an origin located at the center of mass, as pictured in Fig. 1a. The state space vector is thus

$$\begin{aligned} \textbf{x} = \{u, w, q, \theta \} \end{aligned}$$

where *u* and *w* are the body velocities along the $x'-$ and $z'-$axis and $\theta$ and *q* are the pitch angle and its time derivative about the $y'-$axis, respectively. Consequently, the equations of motion read^11,13,26^

1 $$\begin{aligned} \begin{aligned} \dot{u}&= -qw - g\sin \theta + \frac{1}{m_b}\big ( {F_{x'}(\textbf{x}(t), t)} \\&\quad + {F_{x', t}(\textbf{x}(t), t)} + D (\textbf{x}(t), t) \big )\\ \dot{w}&= qu + g\cos \theta +\frac{1}{m_b} \big ( F_{z'}(\textbf{x}(t), t) + F_{z', t}(\textbf{x}(t), t) \big ) \\ \dot{q}&=\frac{1}{I_{yy}} \big ( M_{y'}(\textbf{x}(t), t) + M_{y', t}(\textbf{x}(t), t) \big )\\ \dot{\theta }&= q \end{aligned} \end{aligned}$$

The forcing terms in Eq. (1) are the aerodynamic forces and moments applied to the wing (namely $F_{x'}$, $F_{z'}$, and $M_{y'}$ ) and to the tail ($F_{x', t}$, $F_{z', t}$, and $M_{y', t}$). The whole drag is captured by an extra force *D* that sums contributions due to the body $D_{b}$, the skin friction of the wing (wing profile) $D_{p,w}$, and the skin friction of the tail (tail profile) $D_{p,t}$. These terms are described in detail in the next sections. Importantly, we accounted for the drag acting purely along $x'$ direction, after proving that the projection of the drag forces along $z'$-axis is between two and three orders of magnitude smaller with respect to the aerodynamic forces produced by two other main lifting surfaces. This assumptions holds for the fast forward flight regime that are subject of our study, but such components of drag along $z'$ axis should be accounted for other flight situations.

### Wing model

The bird has two wings. Each wing is a rigid poly-articulated body, comprising the bird arm, forearm and hand, as pictured in Fig. 1b. Each segment is actuated by a joint to induce wing morphing. We refer to^13,15^ for a complete description of this wing kinematic model.

Each joint is kinematically driven to follow a sinusoidal trajectory specified as:

2 $$\begin{aligned} q_{i}(t) = q_{0,i}(t) + A_{i} \sin (\omega t + \phi _{0,i}) \end{aligned}$$

with $\omega = 2 \pi f$ and *f* being the flapping frequency which is identical for each joint, $q_{0,i}$ being the mean angle over a period (or offset), $A_i$ the amplitude, and $\phi _{0,i}$ the relative phase of joint *i*. A complete wingbeat cycle is therefore described through a set of 19 kinematic parameters, including the frequency *f*.

We assume that the wing trajectory is rigidly constrained, and therefore we do not need to explicitly solve the wing dynamics. Under this assumption, the motion generation does not require the computation of joint torques. The model further embeds seven feathers of length $l_{ki}$ in each wing. The feathers in the model have to be considered a representative sample of the real wing feathers. They thus have a limited biological relevance; their number is chosen so as to interpolate the planform satisfactorily and to smoothly capture the morphing generated by the bone movements. These feathers are attached to their respective wing bones via two rotational degrees of freedom allowing them to pitch and spread in the spanwise direction. These two degrees of freedom are again kinematically driven by relationships that depend on the angle between the wing segments^13^. This makes the feathers spreading and folding smoothly through the wingbeat cycle. In sum, the kinematic model of the wing yields the position of its bones and feathers at every time step. This provides a certain wing morphing from which the wing envelope (leading edge and trailing edge) can be computed (see Fig. 1b). From the wing envelope, the aerodynamic chord and the lifting line are computed. The lifting line is the line passing through the quarter of chord, which is itself defined as the segment connecting the leading edge to the trailing edge and orthogonal to the lifting line (Fig. 1b). This extraction algorithm is explained in detail in^15^.

In order to calculate the aerodynamic forces, the angle of attack of the wing profile has to be evaluated. Each wing element defines a plane containing the lifting line and the aerodynamic chord as pictured in Fig. 1c. The orientation of the plane is identified by the orthogonal unit vectors $(\hat{\textbf{e}}_n, \hat{\textbf{e}}_t, \hat{\textbf{e}}_b)$, where $\hat{\textbf{e}}_n$ is the vector perpendicular to the plane and $\hat{\textbf{e}}_t$ is the tangent to the lifting line. To compute the effective angle of attack, the velocity perceived by the wing profile is computed as the sum of the velocities due to the body and wing motion, and the velocity induced by the wake. The first contribution, $\textbf{U}$, accounts for

$$\begin{aligned} \textbf{U} = \textbf{U}_{\infty } - \textbf{v}_{kin} - \textbf{v}_{q}\end{aligned}$$

where $\textbf{U}_{\infty } = u \hat{\textbf{e}}_{x'} + w \hat{\textbf{e}}_{z'}$ is the actual flight velocity, $\textbf{v}_{kin}$ is the relative velocity of the wing due to its motion, and $\textbf{v}_{q}$ is the component induced by the angular velocity of the body *q* and calculated as

$$\begin{aligned} \textbf{v}_{q} = q\hat{\textbf{e}}_{y'} \wedge (\textbf{P}_{i} - \textbf{G})\end{aligned}$$

This velocity vector $\textbf{U}$ defines the angle $\alpha$, as pictured in Fig. 1d.

The second contribution is due to the induced velocity field by the wake, i.e. the downwash velocity $w_{d}$, and acting along the normal unit vector $-w_{d}\hat{\textbf{e}}_n$. The resulting effective angle of attack, $\alpha _{r}$, is thus

$$\begin{aligned} \alpha _{r} = \alpha - \frac{w_{d}}{|\textbf{U}|}\end{aligned}$$

The downwash velocity $w_d$ is computed according to the Biot-Savart law^23^, assuming the wake being shed backwards in the form of straight and infinitely long vortex filaments at each time step of the simulation^13,15^. This quasi-steady approximation is justified a posteriori by ensuring that our reduced frequency, inversely proportional to the unknown airspeed, never exceeds the value of 0.2, below which the effects of time-dependent wake shapes on wing circulation are negligible (e.g. see discussion in^27^). Once the downwash is evaluated, it is possible to evaluate the circulation, and consequently the aerodynamic force and moment acting at the element $P_i$, i.e. $F_{x', i}(\textbf{x}(t), t), F_{z', i}(\textbf{x}(t), t), M_{y', i}(\textbf{x}(t), t)$, as explained in detail in^13^. We use the thin airfoil theory for the estimation of the lift coefficient, with a slope of $2\pi$ that saturates at an effective angle of attack $\alpha _{r}$ of $\pm 15^{\circ }$.

### Drag production by body and wing

The main body and the wings induce drag that should be accounted for in a model aiming at characterizing energetic performance. Body-induced drag is named parasitic because the body itself does not contribute to lift generation, and only induces skin friction and pressure drag around its envelope^28^. The total body drag is

3 $$\begin{aligned} D_{b} = \frac{1}{2}\rho C_{d, b} S_{b}|\textbf{U}_{\infty }|^{2} \end{aligned}$$

where $\rho$ is the air density. We implemented the model described by Maybury^28^ to compute the body drag coefficient $C_{d, b}$. This depends on the morphology of the bird and the Reynolds number *Re* according to

4 $$\begin{aligned} C_{d,b} = 66.6m_{b}^{-0.511}FR_{t}^{0.9015}S_{b}^{1.063}Re^{-0.197} \end{aligned}$$

with $S_{b}$ and $FR_{t}$ are respectively the frontal area of the body and the fitness ratio of the bird, and both of them can be estimated from other allometric formulas i.e.^28,29^.

5 $$\begin{aligned} S_{b}= & {} 0.00813m_{b}^{2/3} \end{aligned}$$

6 $$\begin{aligned} FR_{t}= & {} 6.0799m_{b}^{0.1523} \end{aligned}$$

The Reynolds number $Re = \rho |\textbf{U}_{\infty }| \overline{c} / \mu$ is calculated with the reference length of the mean aerodynamic chord $\overline{c}$, with $\mu$ being the dynamic viscosity. This model is found to be suitable for Reynolds number in the range $10^{4}-10^{5}$^28^. Another source of drag is the profile drag due to friction between the air and the feathers on the wings. It is the sum of the profile drag at each section along the wingspan, i.e.

7 $$\begin{aligned} D_{p,w} = \frac{1}{2} \rho C_{d, pro} \sum _{i=1}^{n} c_{i}|\textbf{U}_{r,i}|^{2} ds_{i} \end{aligned}$$

with $c_{i}$ the chord length, $ds_{i}$ the length of the lifting line element along the wingspan, and $\textbf{U}_{r,i}$ the velocity at the wing section *i* accounting for the body velocity, the kinematics velocity of the wing and the downwash velocity (Fig. 1c,d). We used a value of profile drag of $C_{d, pro} = 0.02$ and this is assumed to be constant over the wingspan and throughout the flapping cycle^30^. In reality, due to the wing motion, this value should be gait dependent. However, the aforementioned assumption has been largely used in previous works^31,32^.

### Tail model

Since the span of the tail is of the same magnitude as its aerodynamic chord, here the lifting line approach cannot be used^23^. Therefore, the tail is modelled according to the slender delta wing theory, as a triangular planform^33^. Its morphology is illustrated in Fig. 1b and characterized by the opening angle $\beta$ and the chord $c_t$. This latter parameter is kept constant, thus the tail span is controlled via $\beta$ from the trigonometrical relationship

$$\begin{aligned} b_{t} = 2c_{t}\tan \frac{\beta }{2}\end{aligned}$$

The main limitation of this framework is the low range of angles of attack ($\alpha _{tail}<5^{^\circ }$) within which it provides accurate results^34^. This limitation is valid in our context of fast forward flight, where the bird flight is straight, horizontal, and the forward velocity *u* is much larger than the vertical one *w*. The velocity component acting on the tail-like surface is

8 $$\begin{aligned} \textbf{U}_{t}(t) = \textbf{U}_{\infty } + \textbf{v}_{ind}^{w\rightarrow t} + \textbf{v}_{ind,b} \end{aligned}$$

where the term $\textbf{v}_{ind}^{w\rightarrow t}$ is the velocity acting on the tail, induced by vortex filament shed by the wing. This velocity is calculated according to Biot-Savart law^23^, and

$$\begin{aligned} \textbf{v}_{ind,b} = q \hat{\textbf{e}}_{y'} \wedge (\textbf{G}-\textbf{N})\end{aligned}$$

is the velocity induced by the body rotation, with $(\textbf{G}-\textbf{N})$ the vector between the center of mass of the body ($\textbf{G}$) and the point of application of the forces on the tail ($\textbf{N}$) taken at two third along the tail chord, as illustrated in Fig. 1b. Furthermore, our tail spread takes $45^{\circ }$ as upper value, i.e. a maximum aspect ratio of 1.65 for our triangular tail. The tail tilt relative to the body is constrained to $0^{\circ }$, so that the tail pitch is the same as the body’s. Furthermore, as the tail kinematic velocity stays far lower than in the wing sections, the stall zone is avoided.

The forces generated by the tail are computed according to^33^

9 $$\begin{aligned} \begin{aligned} F_{x', t}&=\left( \frac{\pi }{4}\rho \alpha _{t} |\textbf{U}_{t}|^{2} b_{t}^{2} \right) \cdot \hat{\textbf{e}}_{x'} \\ F_{z', t}&= \left( \frac{\pi }{4}\rho \alpha _{t} |\textbf{U}_{t}|^{2} b_{t}^{2} \right) \cdot \hat{\textbf{e}}_{z'} \end{aligned} \end{aligned}$$

These forces are applied at point $\textbf{N}$. In addition, adding this tail-like surface introduces another source of drag that needs to be accounted for. This parasitic drag contribution is, according to^33^

10 $$\begin{aligned} D_{p, t}=\frac{1}{2}\rho |\textbf{U}_{t}|^{2} S_{t} C_{D, f} \end{aligned}$$

where $S_{t}$ is the tail planar surface and $C_{D, f}$ the dimensionless friction coefficient.

### Numerical framework

Due to the fact that Eq. (1) are driven by time-varying forces and periodic actuation, they do not converge to steady equilibrium points^11,35^. Instead, the steady condition corresponds to a limit cycle of this model, and its stability can be assessed via dedicated tools. The numerical identification of such a periodic orbit and the characterization of its stability are challenged by the fact that this orbit is unknown a priori. In previous work, we developed a method to achieve both at the same time via a multiple-shooting algorithm. This framework has been released as a Python toolbox called multiflap^13^. It takes as input a set of ordinary differential equations (ODE) like (1), i.e. of the form

11 $$\begin{aligned} \dot{\textbf{x}} = \textbf{v}(\textbf{x}, t, \nu ) \end{aligned}$$

with $\textbf{x}$ being the state variables, $\textbf{v}$ a vector field describing the system dynamics, *t* the time and $\nu$ a set of configuration parameters. A periodic orbit is thus a particular solution of this set of ODE such that

$$\begin{aligned} \textbf{x}(t, \nu ) = \textbf{x}(t+T, \nu )\end{aligned}$$

with *T* being the cycle period. Such a periodic solution, defines a steady flight regime.

The stability of such closed orbits can also be assessed through the long-term response to a perturbation: if the perturbed trajectory converges back to the orbit, then it is stable, and vice-versa. We perform this stability analysis via the Floquet theory^12^. Stability of the set of equations is governed by the eigenvalues of the so-called Floquet matrix $\mathbb {J}$, also known as Floquet multipliers, $\Lambda _{i}$. This Floquet matrix maps perturbations within an infinitesimal sphere around a point of the limit cycle $(\mathbf {x_{0}}, t_{0})$ into an ellipsoid after a time *T* equal to the period of the orbit. Stretching or contracting ratios of the principal axes of this first order transformation are governed by the Floquet Multipliers. Floquet multipliers have the property of being invariant along the limit cycle, whereas the Floquet matrix and its eigenvectors depend on it. Concretely, the Floquet matrix $\mathbb {J}$ can be calculated as the solution of the variational equation:

12 $$\begin{aligned} \begin{aligned} \frac{d\mathbb {J}}{dt}(\textbf{x}_0) \Big |_{t_0}^{t}&= \mathbb {A}(\textbf{x}, t) \mathbb {J}(\textbf{x}_0) \Big |_{t_0}^{t}\\ \mathbb {J} (\textbf{x}_0)\Big |_{t_0}^{t_0}&= \mathbb {I} \end{aligned} \end{aligned}$$

where the matrix

13 $$\begin{aligned} \mathbb {A}(\textbf{x}, t) = \nabla {\textbf{v}(\textbf{x}, t)} | _{\textbf{x}=\mathbf {x^{*}}} \end{aligned}$$

is called the Stability Matrix^36^ and is *T*-periodic on the limit cycle. If the absolute values of all Floquet multipliers $\Lambda _{i}$ are smaller than one, the corresponding periodic orbit is stable. Conversely, if the absolute value of at least one multiplier is larger than one, the corresponding orbit is unstable and the perturbation spirals out of the limit cycle along the corresponding eigendirection(s). This framework provides another important feature, namely the stretching/contracting rate per unit of time, or Floquet exponent, $\lambda _{i}$^36,37^

14 $$\begin{aligned} \lambda _{i} = \frac{1}{T} \ln |\Lambda _{i}| \end{aligned}$$

### Application to steady and level flight

**Figure 2:**
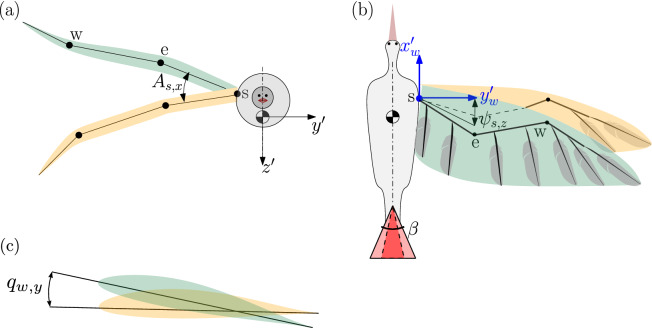
(**a**) Front view of the bird model. The wingbeat amplitude $A_{s,x}$ about the $x'$-axis is the main kinematic parameter governing the wing amplitude of movement. (**b**) Top view of the bird model. The shoulder sweep offset $\psi _{s,z}$ captures the average angle of the arm bone with respect to $x'_{w}$ over the period. The angle $\beta$ captures the magnitude of tail opening. (**c**) Section of the wing. It highlights the wing rotation angle governed by the wrist degree of freedom $q_{w,y}$.

In the present study, we leverage the aforementioned multiple-shooting algorithm, for seeking steady flight regimes within the following parametric space

15 $$\begin{aligned} \nu = (\beta , \psi _{s,z}, A_{s, x}, q_{w,y}) \end{aligned}$$

where $\beta$ is the tail opening angle, $\psi _{s,z}$ is the shoulder sweep offset, $A_{s, x}$ is the wingbeat amplitude, and $q_{w,y}$ is the mean rotation angle of the wing profiles of the forearm about the axis *y*, see Fig. 2. The other parameters defining the wing kinematics are kept fixed to values similar to those reported in^13^.

Previous studies have shown that these four parameters decisively govern the flight regime in bird flapping and gliding modes. The tail opening $\beta$ and shoulder sweep offset $\psi _{s,z}$ influence flight stability, since these are the parameters having a paramount influence on the generation of pitching moment. Then, the shoulder wingbeat amplitude $A_{s,x}$ has a direct impact on thrust production and therefore on airspeed and power consumption^38^. The last parameter $q_{w,y}$ modulates the generation of lift^13,15^.

On top of seeking for steady flight regimes, it is important to identify those corresponding to level flight, i.e. with the bird flying at a constant altitude. This level flight condition thus corresponds to an average mean vertical velocity being equal to zero over the period, in a fixed reference frame. Figure 1 shows that this instantaneous vertical velocity can be computed as

16 $$\begin{aligned} W_{ff} = -\dot{Z} = u\sin {\theta } - w\cos {\theta } \end{aligned}$$

Concretely, satisfying the level flight condition isolates a three-dimensional manifold within the four-dimensional parametric space of Eq. (15). Finding this manifold is done by searching the value of the parameter $q_{w,y}$ that corresponds to level flight for all possible combinations of the three other parameters $\beta$, $\psi _{s,z}$ and $A_{s,x}$. In other words, we report here only the limit cycles that belong to the manifold satisfying

17 $$\begin{aligned} \left| \overline{{W}_{ff}}({\beta }, {\psi _{s,z}}, {A}_{s, x},q_{w,y}) \right| < \epsilon \end{aligned}$$

with $\epsilon = 5 \times {10}^{-3}$ ms$^{-1}$, which corresponds to a maximum vertical deviation of 1 mm per flapping cycle.

### Power consumption and cost of transport

Each limit cycle corresponds to a particular flapping gait with its own mechanical power consumption and the corresponding cost of transport.

Since inertial power for accelerating and decelerating a wing is neglected, the actuation power produced by the wing joints is exactly equal to the power transferred by this wing to the environment, i.e.

18 $$\begin{aligned} P_{act}(t) = \sum _{i=1}^{n}\textbf{F}_{aero,i}(t) \cdot (-\textbf{v}_{kin, i}(t)) \end{aligned}$$

where $\textbf{v}_{kin, i}(t)$ is the velocity of the lifting line computed at the discretized point $P_i$ and time *t*, and ${F}_{aero,i}(t)$ is the corresponding aerodynamic load on the wing element *i*, computed by the quasi-steady lifting line model. The mean power consumption over one flapping period is thus

19 $$\begin{aligned} \overline{P}_{act} = \frac{1}{T}\int _{0}^{T} P_{act}(t) dt \end{aligned}$$

Another important metric to assess locomotion performance is the so-called Cost of Transport (CoT), i.e. a dimensionless ratio equal to the mechanical work produced by the actuators to transport a unit of body weight across a unit of distance^39^. Here, it is thus defined as

20 $$\begin{aligned} CoT = \frac{\overline{P}_{act}}{mg\overline{|\textbf{U}_{\infty }|}} \end{aligned}$$

with $\overline{|\textbf{U}_{\infty }|}$ being the magnitude of the flight speed of the corresponding limit cycle, averaged over one period.

### Parametrization of bird morphology and wing kinematics

The above framework and concepts are here embodied in a model of the northern bald ibis (*Geronticus eremita*). This species does not only display features at the origin of possible conflict between energetic and stability demands (continuous flapping propulsion and long migratory flights), but it is often studied by biologists because of its endangered status. The length of bones and feathers are set up accordingly. To the best of our knowledge, the precise wingbeat kinematics of this particular bird have not been reported in the literature. Consequently, we follow the same approach as in^13^, consisting in scaling up the detailed kinematic pattern of another bird^40^ to the morphology of ours. This scaling process, and the parameter tuning are extensively detailed in the Supplementary Material S1. The morphological and kinematic parameters used to describe the bird are reported in Table S1 and the resulting flapping gait is illustrated in Figs. S1, S2 and S3.

This study is performed within the following parametric space: tail opening $\beta \in [0^{\circ },45^{\circ }]$, wingbeat amplitude $A_{s,x} \in [29^{\circ },45^{\circ }]$ and sweep offset $\psi _{s,z} \in [9^{\circ },15^{\circ }]$. The parametric space is meshed with an uniform grid spaced along $\psi _{s,z}$ and $A_{s,x}$ with a step size of $0.5^{\circ }$, and a step size of $1^{\circ }$ along $\beta$. This resulted in 19,734 possible flight configurations. In the results, we report all solutions satisfying Eq. (17), with the addition of two exclusion criteria. First, we excluded limit cycles that do not correspond to fast forward flight. In^41^, this corresponds to flying modes 4 and 5 and requires the body pitch angle to stay close to the horizon tail configuration. Concretely, we excluded from the results limit cycles corresponding to a mean body pitch angle larger than $6^{\circ }$ in absolute value. Second, we excluded limit cycles corresponding to biologically incompatible kinematics. This was implemented by excluding limit cycles with a mean rotation angle of the wing $q_{w,y}$ larger than $12^{\circ }$ in absolute value. Indeed, remembering that the related amplitude of this joint was fixed to $30^{\circ }$ (Table S1 in the Supplementary Material), this criteria excluded solutions corresponding to geometrical rotation of the forearm larger than $\pm 42^{\circ }$ in absolute value, which we considered to be not physiologically consistent.

## Results

In this section, we report the results of the systematic exploration of the gait parametric space. First, the locus of the solutions is reported, i.e. the set of parametric values for which a limit cycle has been identified. Next, three representative limit cycles are analysed in detail: the first one with a completely furled tail ($\beta = 0$), and the both other ones with an open tail ($\beta = 40^{\circ }$). Finally, these solutions are assessed in terms of energetic expenditure, quantified by the CoT.

### Locus of solutions

**Figure 3:**
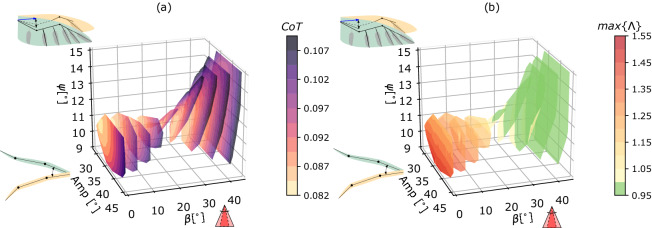
Locus of the steady and level solutions in the gait parameter space. Coloured surfaces are representing slices of this locus, at every $5^{\circ }$ of tail opening. (**a**) Cost of Transport. (**b**) Stability indicator captured via the largest Floquet multiplier. Unstable limit cycles are represented with different shades of yellow-to-red, and stables ones are in green; the transition appears around $\beta = 25 ^{\circ }$ (Color figure online).

Among the 19,734 possible parametric configurations, our algorithm detected 5604 steady level limit cycles which do not violate the exclusion criteria listed above. The locus of these identified solutions is pictured in Fig. 3.

Figure 3a shows the CoT at equally-spaced planes of tail opening angle $\beta$, projected in the parameter space. The CoT progressively increases as the tail spreads out. It further displays a higher gradient with respect to both other parameters for a given opening angle. This reveals that this cost of transport is sensitive to the kinematic parameters governing wing movements. Figure 3b illustrates the stability transition that occurs as a function of the tail opening. The bifurcation point happens for a value around $\beta = 25^{\circ }$. The largest Floquet multipliers in the stable region are however never smaller than about 0.96, corresponding to a largest stable Floquet exponent being equal to (see Eq. (14))

21 $$\begin{aligned} \lambda _{max} = -0.16 s^{-1} \end{aligned}$$

Solving Eq. (14) for *t*, the time taken for halving a perturbation is therefore

22 $$\begin{aligned} t_{half} = 4.2 s \end{aligned}$$

which corresponds to about 17 flapping periods.

As revealed by the quasi-horizontal stripes of uniform colours in Fig. 3b, the shoulder amplitude has a marginal effect on stability—because of its marginal role on the distribution of nose up/down pitching moment—in contrast to the tail opening and sweep angle.

### Comparison between furled and open tail solutions

In this section, three representative limit cycles are further investigated: one corresponding to a tail completely furled ($\beta =0$) and the other ones to a tail opening of $\beta =40^{\circ }$. These reference limit cycles are selected to have the same resulting forward flight velocity, i.e. 14 ms$^{-1}$. The whole set of corresponding parameters is reported in Table 1.

**Table 1:** Parameters for the three representative limit cycles studied in more detail, corresponding to one unstable and two stable flight regimes, respectively, at a forward flight velocity of 14 ms$^{-1}$.

	(a)	(b)	(c)
$\beta [^{\circ }]$	0	40	40
$\psi _{s,z} [^{\circ }]$	10.5	14.5	13.5
$A_{s,x} [^{\circ }]$	39.5	42	44
$q_{w,y} [^{\circ }]$	2.3	$-5.5$	$-9.67$

**Figure 4:**
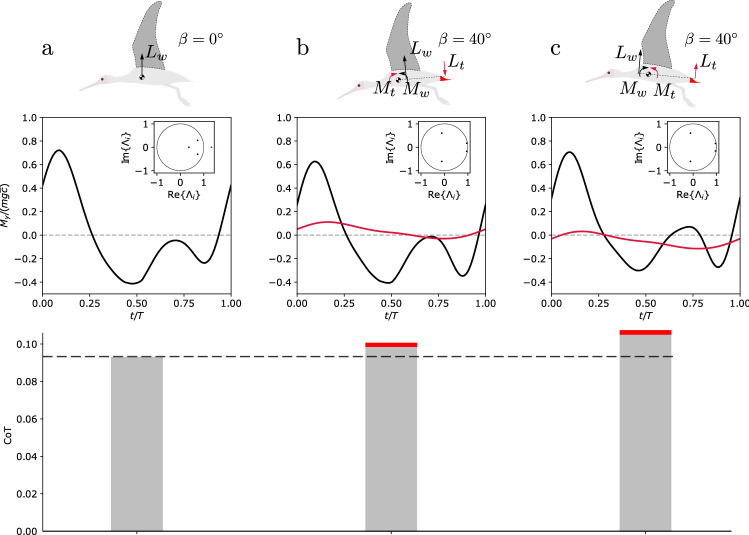
Characterization of three representative limit cycles: one with furled tail (**a**), and two with a tail opened with an angle $\beta = 40^{\circ }$ (**b**, **c**). The upper panel represents the free-body diagram of the three different flight configurations. Case (a): The pitching moment is only due to the wing movement, and averages at zero. This flight regime is characterized by an unstable mode, highlighted by an eigenvalue larger than 1 (inset middle panel). The bottom panel gives the CoT for this flight configuration. Case (b): The average pitching moment $M_{w}$ due to the wing lift ($L_{w}$) is negative (nose down) and the average moment due to the tail lift, $M_{t}$, is positive (nose up). This solution is stable as all the eigenvalues are smaller than 1 in absolute value (inset middle panel). The CoT is quantified in the bottom panel, and with the contribution due to dissipative forces acting on the tail being highlighted in red. Case (c): The average pitching moment due to the wing lift ($L_{w}$) is positive (nose up) and the average moment due to the tail lift is negative (nose down). This solution is also stable as all the eigenvalues are smaller than 1 in absolute value (inset middle panel).

The free-body diagram of these configurations is illustrated in Fig. 4-top panel. The actual pitching moment characterizing the limit cycle solutions is reported in Fig. 4-middle panel. In case (a) (furled tail), the wing must guarantee that the time integral of moment over one flapping cycle is equal to zero in order to assure the existence of a limit cycle. Both open-tail configurations corresponds to different configurations of momentum equilibrium. In case (b), the wing contributes for nose-down moment (on average) balanced by the nose-up moment (on average) of the tail. On average, the body pitch angle over one flapping cycle is about $-0.2^{\circ }$. Conversely, in the case (c), the wing contributes for nose-up moment (on average) balanced by the nose-down moment (on average) of the tail. On average, the body pitch angle over one flapping cycle is about $1.3^{\circ }$. All these configurations exhibit a similar limit cycle regarding the phase space trajectory, although the former has one unstable mode in pitch stability governed by a Floquet multiplier equal to $\Lambda =1.33$. The open tail cases are both stable since their largest multiplier has a magnitude equal to about $\Lambda =0.96$. All multipliers are pictured in the inset plot of the middle panel of Fig. 4.

The power required to achieve level flight in the furled tail case (a), averaged over one wingbeat cycle, is equal to 15.4 W. In case (b), it is equal to 16.7 W with a contribution to the tail-parasitic drag of about 0.4 W, while in case (c) it is equal to around 17.9 W with a power dissipated by drag-induced forces in the tail of about 0.4 W. This power assessment is pictured adimensionally in the bottom panel of Fig. 4, where the red stripes corresponds to the power dissipation from the tail. There is thus a trade-off between robustness to perturbations—characterized by passive stability—and performance—characterized by the required mechanical power.

**Figure 5:**
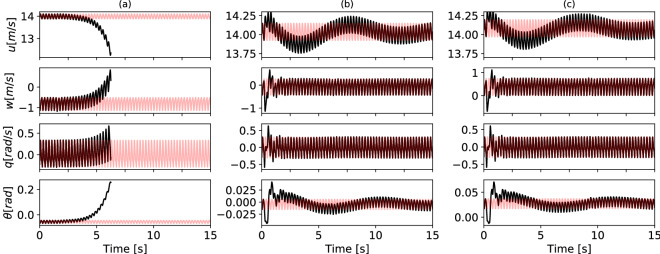
Dynamic response of the three representatives limit cycles, to a Gaussian-like upward gust. Case (**a**) Separation of the perturbed solution along the unstable eigendirection (black) from the periodic orbit. Case (**b**) and (**c**) Passively stable response. After a transient, the perturbed trajectory (black) tends to converge back to the level steady flight condition (red) (Color figure online).

These three representative limit cycles have been perturbed by an upward gust along the local $z'$-axis. The gust is modeled as a Gaussian signal $w_{g}$ in the form:

23 $$\begin{aligned} w_{g}(t)= -w_{0}\exp \Big (-\frac{1}{2} \Big ( \frac{t-t_p}{\sigma }\Big )^{2} \Big )\hat{\textbf{e}}_{z'} \ \forall t > 0 \end{aligned}$$

with $t_{p} = 0.25s$ and $\sigma =0.05s$. The intensity of the gust was tuned in order to observe comparable effects in phase space. In the unstable case (a), $w_{0} = 0.1$ ms$^{-1}$, whereas in the stable cases (b) and (c) $w_{0} = 1$ ms$^{-1}$. The dynamic response of these three configurations is captured by the black solid lines in Fig. 5. Figure 5a shows a quick separation from the limit cycle condition (red curves), driven by the unstable Floquet multiplier. Figure 5b,c show a passively stable response to the perturbation as all the Floquet multipliers are smaller than 1 in both cases. This attraction is dominated by two characteristic times, depending on the absolute value of the Floquet multipliers. A rapid response happens for *w* and *q*, while a slower response resembling a phugoidal mode^26,42^, with period of about 8 s, characterizes the trends of *u* and $\theta$.

### Trade-off between CoT and flight stability

**Figure 6:**
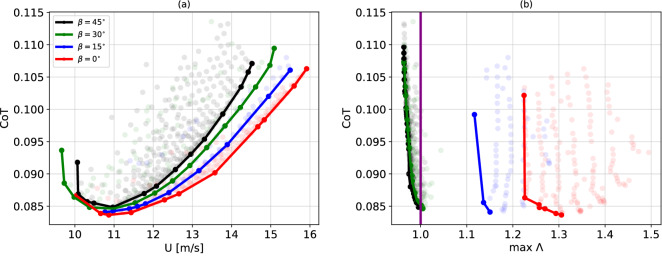
Trade-off between CoT and stability. (**a**) Lower envelope of the CoT as a function of the forward flight velocity of four evenly-spaced $\beta$-planes. We report all the possible solutions, from which the lower envelope is extracted, with transparent points, coloured accordingly with the respective tail opening. (**b**) Pareto front of the CoT as a function of the largest Floquet multiplier of four evenly-spaced $\beta$-planes. We report all the possible solutions, from which the Pareto front is extracted, with transparent points, coloured accordingly with the respective tail opening. The stability transition is highlighted with the purple vertical line (Color figure online).

Figure 6 further illustrates a trade-off between stability and CoT associated with tail spread. Figure 6a, illustrates the lowest achievable CoT as a function of the forward flight velocity. This is represented at four different values of tail opening, namely $\beta = [0, 15, 30, 45]$deg. The minimum of the four curves is around 0.085 and corresponds to forward flight velocities of approximately 11 ms$^{-1}$. The steepness of the curve at increasing velocities monotonically increases with the tail opening. For the same values of $\beta$, Fig. 6b reports the Pareto front of the largest Floquet multiplier $\Lambda$ and CoT. This front captures the optimal solutions for which one of these features could not be more favorable without negatively affecting the other. The transition between stable and unstable flight regimes is highlighted by the vertical purple line.

## Discussion and conclusion

We performed a four-dimensional bifurcation study in the parametric space of flapping gaits. Our numerical analysis highlights the existence of two sets of solutions as a function of the tail opening, with their respective stability properties.

Figure 3 shows that steady level flight can be achieved for a large set of parameter combinations. Such combinations have to balance the pitching moment generated by the wing and the tail. This condition is mainly driven by the sweep offset of the wing and the angle of tail opening. Both of these parameters indeed modulate the distribution of nose-up and nose-down moment, and thus play a fundamental role in the limit cycle stability. The shoulder amplitude only marginally affects stability, confirming the results reported in^13^. This is due to the fact that it does not have an effect in moving the aerodynamic forces forward or backwards—on average—with respect to the center of mass, and thus in altering the pitching moment distribution.

Two profiles of pitching moments that guarantee a passively stable limit cycle have been found. One configuration is similar to those guaranteeing static pitch stability in aircraft and in bird gliding^5,26^, i.e. with the wings generating nose-down moment on average, and the tail generating nose-up moment on average (Fig. 4b). The second configuration guaranteeing stable limit cycles produces a nose-up moment on average with the wing, and nose-down moment with the tail. These two stable configurations, previously described in gliding^8^, are shown here to also apply to flapping of medium to large-size birds. In^3^, Smith stated a biological intuition that birds lost the capacity to rely on passively stable configurations while developing sensory-driven neural circuitries to actively control their flight over the course of evolution. However, this has been recently challenged for gliding flight^5,7^. It was shown that, in gliding regimes, birds can modulate the elbow sweep to achieve passive stability. Here, we extend this promising thesis to flapping regimes, showing that passive stability can also be achieved with appropriate wing kinematics, and tail opening. However, opening the tail comes with an important additional energetic cost. A power analysis revealed that this additional energetic cost is due both to overcome the extra drag produced by the tail, but also to the intrinsic efficiency of the adopted wing kinematics leading to the same flight velocity. Figure 4, indeed shows that the extra power required to operate with the open tail conditions, is not only due to drag forces produced by the tail itself, but also to a more costly kinematics adaptation of the gait to level the flight.

The stability of these three conditions has been analysed under a Gaussian-like gust perturbation. The unstable case shows a quick separation from the limit cycle condition. In the stable solutions, two characteristic times appear: a fast mode of response, affecting the variable *w*, *q*, and a slow phugoid-like mode that affects *u* and $\theta$.

The energetic cost of flapping flight has been repeatedly estimated in literature either by measurements of metabolic proxies such as oxygen consumption and gas exchange (power input) or, directly, via analyses of muscle and wake (power output). Out of comparative analyses, optimal speeds require metabolic costs of transport of the order of $10^0$ if normalised as in Eq. (20). Nevertheless, it remains challenging to quantitatively convert these measurements into power outputs, i.e. the power injected into the airflow. This is because energy conversion efficiency represents a debatable quantity. It is however believed that, for birds, efficiency lies between 10 and 20% (see discussion in^30,43^). Therefore, our model estimates must be increased accordingly. All considered, we show optimality at cost of transport of about $10^{-1}$, which, multiplied by a factor of 5–10, reconduces to previously mentioned observations. Interestingly, according to the allometric formula obtained in^44,45^ by analysing a large number of species, the optimal cost of transport is expected to scale with body mass as

$$\begin{aligned} \text {CoT}=0.109M^{-0.122},\end{aligned}$$

which returns in our case CoT=0.107, very close to the optimal values shown in Fig. 6. The trade-off between stability and energetic performance —associated with tail spread—is highlighted in Fig. 6. The lower envelope of the CoT shows a monotonic increase of curvature with the tail opening. For forward flight velocities of 14 ms$^{-1}$, the saving in terms of CoT between a furled tail configuration and a full open tail is of about $10\%$. This is comparable to the energetic advantage drawn from formation flight with respect to solo flight, according to^46^. CoT curves with small curvatures are crucial for long range flights, as it allows to modulate the velocity at a lower energetic cost. Figure 6b shows the Pareto front of the CoT with respect to the largest Floquet multiplier. The Pareto front of the stable solutions (black and green) is very steep, suggesting that little advantages of stability gain come with a disproportionate energetic cost. We infer the existence of a close interplay between stability and energetic cost of flapping for medium to large size birds in steady flight. Indeed, whereas the absolute minimum CoT only marginally changes as a function of the tail opening, its large variation with respect to the forward velocity does vary as a function of this angle. Put differently, the typical U-shaped curve characterizing the CoT as a function of the forward flight velocity is found at each tail opening angle, but its asymptote is smaller for smaller angles.

In this study, we focused on wing and tail contributions to the longitudinal dynamics. To increase the model fidelity, it will be necessary to account for other morphological and biological elements that may contribute to stabilize the flight. These would need a substantial adaptation of the equations of motion used in the current work. Moreover, in the current version of our model the wings are assumed to be rigid and no kinematic adaptation is implemented to react to a perturbation and thus the potential implication on the stability. This should be relaxed in a more bio-compatible version of the model, that should account for the intrinsic joint compliance due to actuation by muscle-tendon units. This will definitively influence the dynamics of the response to perturbation such as during gust alleviation. Including these effects would necessarily imply to account for the wing dynamics—and the related inertial effects—in the body model. Given the complexity of the wing poly-articulation, this would require a substantial extension of our framework, and a new derivation of the equations of motion which are left as perspectives of the present work. Aeroelasticity along the wingspan, descending from realistic feathers and tendons mechanical response to aerodynamic loads should have a similar influence on stability and will be studied in future work^47^. Similarly, a parametric analysis on the wing flexion during upstroke—a well-observed mechanism used by birds and bats to reduce the exposed wing surface and minimise the drag^41^—has not been tackled in the present study, but could reveal important insights from an energetic point of view.

We used a carefully formulated framework to study steady flight stability, i.e. Floquet theory combined with a multiple-shooting algorithm. We concluded that in spite of the gain in stability, having a tail-like surface induces an increase of steepness of the CoT with respect to the forward flight velocity, that limits the authority of the bird to modulate the flight speed. This suggests an explanation for the field observation that birds flap with furled tails during long flights^20^, i.e. that a loss of dynamic stability might be traded-off in exchange for the freedom of modulating velocity at lower energetic expenditure. This might prove to be a crucial factor, for instance, in seasonal migrations, where the time of arrival at foraging, breeding and wintering sites is naturally constrained by environmental factors such as daylight duration, food availability or social reasons. However, our results show that birds still have the authority to select passively stable modes—i.e. with an open tail—that may prevail in certain circumstances, such as flying while sleeping^48^.

Our present analysis concerns flight regimes characterised by high aspect ratio wings, moderate reduced frequency, and continuous flapping gaits. Those traits are generally shared among a few migratory species, e.g. cranes, ibis, and geese. A wider generalization to smaller bird scales of our theoretical conclusions would require including features that are not explored in this work, such as different wing kinematics, more tailored aerodynamic models capturing wake unsteadiness and leading vortex separation among others. Despite these inevitable modifications at the underlying physical level, the kernel of the proposed framework, i.e. Floquet dynamic stability applied to equations of motion forced by aerodynamics, proves to be a highly valuable option for the study of morphology and gait tuning in avian flight.

## Supplementary Information

Supplementary Information.

## Data Availability

All data generated or analysed during this study are included in this published article, and available as supplementary files.
