# Supplementary material for: On the role of tail in stability and energetic cost of bird flapping flight
Source: Sci Rep. 2022 Dec 31;12:22629. doi: 10.1038/s41598-022-27179-7 (PMC9805461; doi:10.1038/s41598-022-27179-7)
Supplement: Supplementary file 1 — Supplementary Information. [file 41598_2022_27179_MOESM1_ESM.pdf]

Supplementary material

On the role of tail in stability and energetic cost of bird flapping flight

Gianmarco Ducci, Gennaro Vitucci, Philippe Chatelain, Renaud Ronsse

on December 9, 2022

S1 Table S1 - Morphological and kinematic parameters of the northen bald ibis

The bird parameters to capture the morphology of the northern bald ibis are reported in Table S1. The data related to the bird morphology are listed in Table S1(a). The parameters governing the wing kinematics after solving Equation 2 are reported in Table S1(b).

(a) Parameters describing the bird morphology.

Body	
Mass (m_b), $[kg]$	1.2
Moment of Inertia (I_{yy}), $[kg \cdot m^2]$	0.1
**Wing**	
Wingbeat frequency f, $[Hz]$	4
Wingspan (b), $[m]$	1.35
Mean aerodynamic chord ($\bar{c}$), $[m]$	0.15
Arm bone length (l_a), $[m]$	0.134
Forearm bone length (l_f), $[m]$	0.162
Hand bone length (l_h), $[m]$	0.084
**Feathers**	
Primary feather 1 (l_{k1}), $[m]$	0.25
Primary feather 2 (l_{k2}), $[m]$	0.275
Primary feather 3 (l_{k3}), $[m]$	0.25
Secondary feather 1 (l_{k4}), $[m]$	0.225
Secondary feather 2 (l_{k5}), $[m]$	0.2
Secondary feather 3 (l_{k6}), $[m]$	0.175
Secondary feather 4 (l_{k7}), $[m]$	0.15
**Tail**	
Tail chord (c_0), $[m]$	0.25
Tail opening (β), $[°]$	β^*

(b) Kinematic parameters capturing the trajectory of the wing joints, according to Equation (2).

Joint	q_0[deg]	A[deg]	ϕ[deg]
Shoulder y	11.5	0.8	-90
Shoulder x	0	$A^*_{s,x}$	180
Shoulder z	$\psi^*_{s,z}$	14	90
Elbow z	10	10	-90
Wrist y	$q^*_{w,y}$	30	-90
Wrist z	-30	30	90

Table S1: Numerical parameters used in the simulations. Parameters highlighted with an asterisk * are those being varied in the parametric study

The dimension of the wing, namely the length of the humerus, ulna, and metacarpals, have been selected via previous results shown in literature [1, 2, 3, 4]. The choice of the length of these segments relies on the linear regression in $log - log$ plot comparing the available data as a function of the mass of the species collected.

As stated in the manuscript, the kinematics is governed by sinusoidal trajectories in the form

$$q_i(t) = q_{0,i}(t) + A_i \sin(\omega t + \phi_{0,i}) \quad (1)$$

Sinusoidal trajectories are a simplistic approximation of actual kinematics. However, despite possible inaccuracies, this choice allows us to drastically reduce the number of parameters involved in the problem, but yet permits to reproduce flapping gaits exploiting all its features (sweep, folding, etc.).

The kinematic parameters of Equation 1 were tuned as follows. We assumed the bird having its largest wing extension in the downstroke phase, and its minimum extension during the upstroke [5, 6]. This wing extension/folding is regulated via the kinematics equation about the $z'-$axis, and thus imposing the relative phase of the three joints as

$$\phi_{s,z} = \phi_{w,z} = 90^\circ$$

and

$$\phi_{e,z} = -90^\circ$$

.

Moreover, another assumption is to set the wing maximum extension around the middle downstroke, namely at time $t = 0$. The maximum extension implies the three wing segments being aligned, and so the elbow angle $q_{e,z}(0) = 0$ and $q_{w,z}(0) = 0$, and thus

$$A_{e,z} = q_{0,e,z}$$

and

$$A_{w,z} = -q_{0,w,z}$$

. These values have been set in order to have a qualitative tip trajectory that resembles to the ones shown by Tobalske [5]. The values of $A_{s,z}$ is kept constant to a value of 14°, which suffice enough nose-up and nose-down moment to assure limit cycle conditions in absence of the tail surface.

As last, the relative phase of the joints governing the wing pitch, and thus the rotation of the wing profiles about the $y-$axis is modeled in such a way the wing is pitching up during the upstroke, and pitching down during the downstroke, in order to reduce the variations of the angle of attack during the whole flapping period. From a mathematical point of view, this implies setting a phase of

$$\phi_{i,y} = -90^\circ$$

where i represents the shoulder and wrist joints.

The resulting kinematics — and the wingtip trajectory — are reported for the three views of the bird in Figure S1, S2, S3.

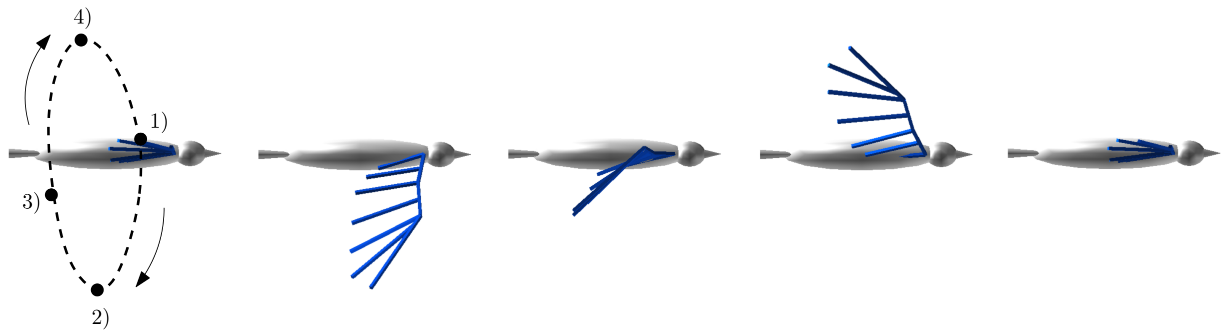

Figure S1: Lateral view of the wingbeat kinematics over one flapping period. The dashed line corresponds to the wing tip trajectory. 1) Middle downstroke. 2) Full downstroke. 3) Middle upstroke. 4) Full upstroke.

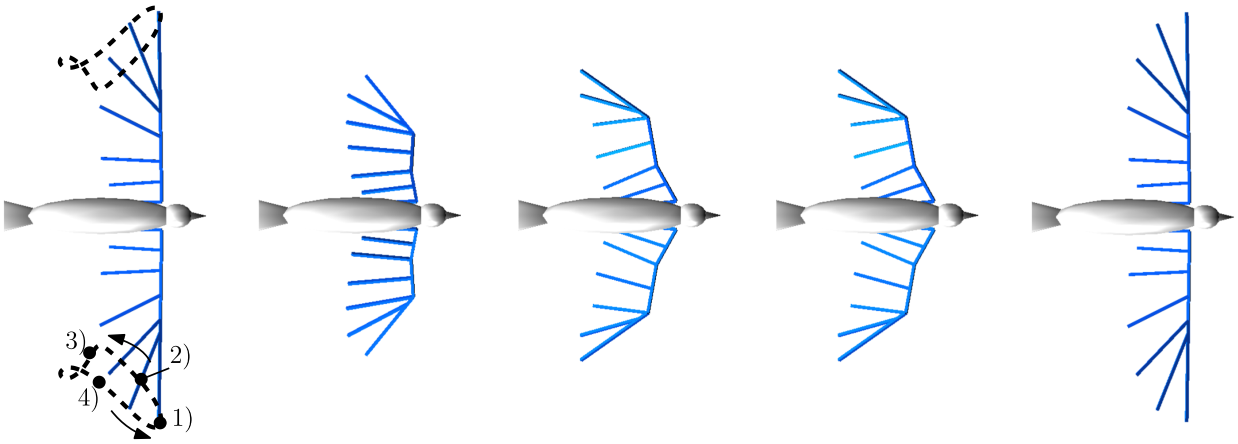

Figure S2: Top view of the wingbeat kinematics over one flapping period. The dashed line corresponds to the wing tip trajectory. 1) Middle downstroke. 2) Full downstroke. 3) Middle upstroke. 4) Full upstroke.

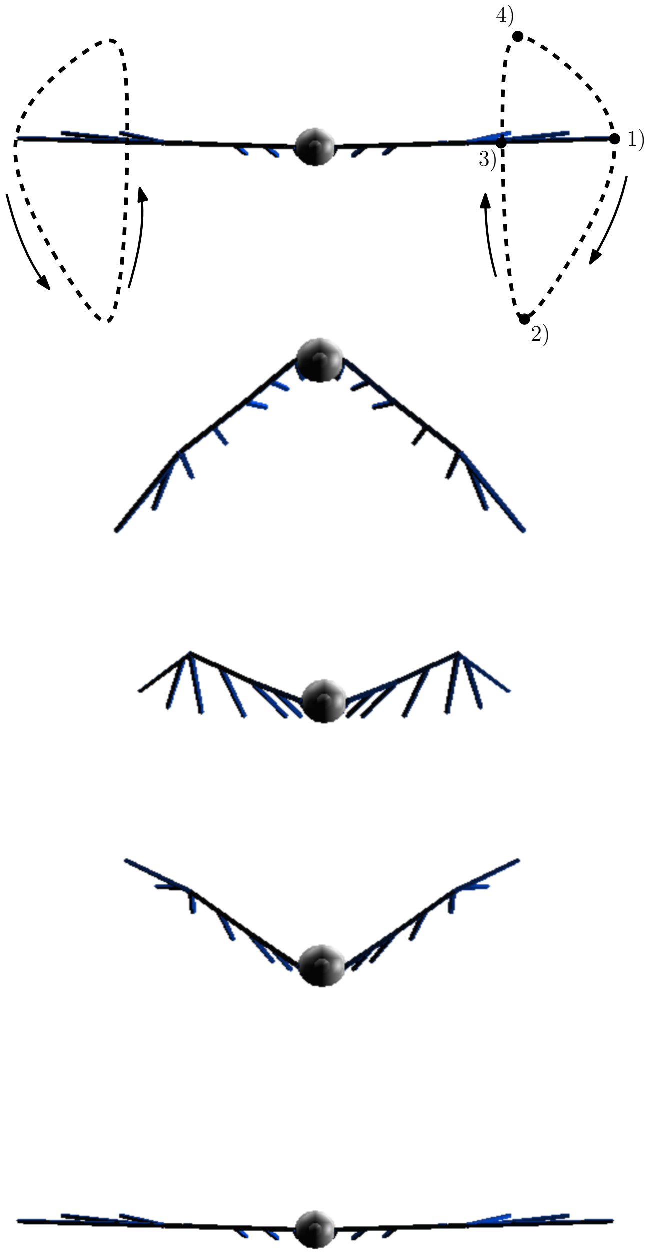

Figure S3: Frontal view of the wingbeat kinematics over one flapping period. The dashed line corresponds to the wing tip trajectory. 1) Middle downstroke. 2) Full downstroke. 3) Middle upstroke. 4) Full upstroke.

S2 Additional files

In order to reproduce all the Figures presented in the paper, we provide the available data of the simulations, as well as the `Python` scripts. The file are are zipped in the `supplementary_files` folder, and the listing is here reported:

- `dataset.csv`: dataset of the detected limit cycles in `.csv` form. It is structured as follows:

β [°]	$\psi_{s,z}$ [°]	$A_{s,x}$ [°]	CoT []	U_{ff} [m/s]	W_{ff} [m/s]	P [W]	Λ_{max}[]	$q_{w,y}$ [°]	θ [rad]	M_w [Nm]	M_t [Nm]

  where:

  - β: tail opening;
  - $\psi_{s,z}$: sweep offset of the shoulder
  - $A_{s,x}$: wingbeat amplitude;
  - CoT: cost of transport;
  - U_{ff}: resulting forward flight velocity in the inertial frame;
  - W_{ff}: resulting vertical velocity in the inertial frame;
  - P: power;
  - Λ_{max} : largest Floquet multiplier;
  - $q_{w,y}$: resulting elbow offset;
  - θ: resulting body pitch angle;
  - M_w: moment of the wing;
  - M_t: moment of the tail.

- `tail_x_sweep_y_SAz_z`: Folders. They contain the limit cycle solutions, the gust simulations, and the aerodynamic moments to reproduce the three cases reported in Section *Comparison between furled and open tail solutions*. The nomenclature is named accordingly to each study case.
- `figure_3.py`: `Python` script. It reads `dataset.csv` and produces Figure 3.
- `figure_4.py`: `Python` script. It reads the three limit cycles solutions, and produces Figure 4.
- `figure_5.py`: `Python` script. It reads the gust simulations, and produces Figure 5.
- `figure_6.py`: `Python` script. It reads `dataset.csv`, and produces Figure 6.

All the attached files are standalone and commented accordingly to help the readability. We remind the user to check the proper `path-to-file` in order to read the different dataset on the local machine.

Bibliography

[1] Simons, E. L., "Forelimb skeletal morphology and flight mode evolution in pelecaniform birds," *Zoology*, Vol. 113, No. 1, 2010, pp. 39–46.

[2] Wang, X., McGowan, A. J., and Dyke, G. J., "Avian wing proportions and flight styles: first step towards predicting the flight modes of Mesozoic birds," *PLoS One*, Vol. 6, No. 12, 2011, pp. e28672.

[3] Field, D. J., Lynner, C., Brown, C., and Darroch, S. A., "Skeletal correlates for body mass estimation in modern and fossil flying birds," *PloS one*, Vol. 8, No. 11, 2013, pp. e82000.

[4] Pennycuick, C. J., *Modelling the flying bird*, Elsevier, 2008.

[5] Tobalske, B. and Dial, K., "Flight kinematics of black-billed magpies and pigeons over a wide range of speeds," *Journal of Experimental Biology*, Vol. 199, No. 2, 1996, pp. 263–280.

[6] Parslew, B. and Crowther, W. J., "Simulating avian wingbeat kinematics," *Journal of Biomechanics*, Vol. 43, No. 16, 2010, pp. 3191–3198.
